# Assessment of Autism Spectrum Disorder in Deaf Adults with Intellectual Disability: Feasibility and Psychometric Properties of an Adapted Version of the Autism Diagnostic Observation Schedule (ADOS-2)

**DOI:** 10.1007/s10803-021-05203-5

**Published:** 2021-07-28

**Authors:** D. Holzinger, C. Weber, S. Bölte, J. Fellinger, J. Hofer

**Affiliations:** 1 Forschungsinstitut für Entwicklungsmedizin, Johannes Kepler Universität Linz, Linz, Austria; 2 Institut für Sinnes- und Sprachneurologie, Konventhospital Barmherzige Brüder, Seilerstätte 2, 4021 Linz, Austria; 3 Institut für Sprachwissenschaft, Karl-Franzens-Universität Graz, Graz, Austria; 4 Institut für Inklusive Pädagogik, Pädagogische Hochschule OÖ, Linz, Austria; 5 Department of Women’s and Children’s Health, Center of Neurodevelopmental Disorders (KIND), Centre for Psychiatry Research, Karolinska Institutet & Stockholm Health Care Services, Region Stockholm, Stockholm, Sweden; 6 Child and Adolescent Psychiatry, Stockholm Health Care Services, Region Stockholm, Stockholm, Sweden; 7 Curtin Autism Research Group, Curtin School of Allied Health, Curtin University, Perth, WA Australia; 8 Abteilung für Sozialpsychiatrie der Universitätsklinik für Psychiatrie und Psychotherapie, Medizinische Universität Wien, Vienna, Austria; 9 Abteilung für Pädiatrie I, Medizinische Universität Innsbruck, Innsbruck, Austria

**Keywords:** Autism spectrum disorder, ADOS-2, Deaf, Sensory impairment, Intellectual disability, Diagnosis, Reliability

## Abstract

This study describes the adaptation of the autism diagnostic observation schedule (ADOS-2) to assess autism spectrum disorder (ASD) in adults with intellectual disability (ID) and hearing loss who communicate primarily visually. This adapted ADOS-2 was applied to residents of specialized therapeutic living communities (n = 56). The internal consistency of the adapted ADOS-2 was excellent for the Social Affect of modules 2 and 3 and acceptable for Restricted and Repetitive Behaviors subscale of module 2, but poor for module 3. Interrater reliability was comparable to standard ADOS-2 modules 1–3. Results suggest that autism symptoms of deaf adults with ID can be reliably identified by an adapted ADOS-2, provided adequate expertise in deafness, ID, ASD and proficiency in signed language by the administrator.

## Introduction

Autism spectrum disorder (ASD) is a neurodevelopmental condition of heterogeneous origin and phenotypic expression of social communication and interaction difficulties along with repetitive, restricted behaviors (American Psychiatric Association, 2013). The diversity of individual presentation with autism symptoms can pose diagnostic and intervention challenges (Jeste & Gschwind, 2014). ASD diagnosis is exclusively behaviorally-defined, making the “best clinical judgment of experienced clinicians” the diagnostic gold standard (Volkmar et al., 2014; National Collaborating Centre for Mental Health (UK), 2012). However, even for experienced clinicians a multidisciplinary, consensus-based diagnostic assessment including standardized instruments is recommended and improves the accuracy of ASD diagnosis (Guthrie et al., 2013; Kim & Lord, 2012). In this context the autism diagnostic observation schedule 2nd edition (ADOS-2; Lord et al., 2012) is considered the “gold-standard”, allowing clinician led observation and evaluation of ASD defining symptoms in the course of structured playful and interview-based interactions. The ADOS-2 is composed of five modules: the Toddler Module for children aged 12–30 months without phrase speech, Module 1 (M1) for children aged > 30 months who do not show phrase speech, Module 2 (M2) for children with phrase speech who are not verbally fluent, Module 3 (M3) for children and young adolescents with fluent language, and Module 4 (M4) for older adolescents and adults with fluent language.

Studies have reported satisfactory to excellent diagnostic validity of the ADOS in different settings (Bölte & Poustka, 2004; de Bildt et al., 2009; Gotham et al., 2007, 2008; Zander et al., 2015). Sensitivity and specificity of the ADOS vary across clinical and research settings as a result of differences in examiner skills and testee related factors (Gotham et al., 2007). The psychometric properties of the ADOS-2 are less well established in adult autistic populations, for autistic adults with psychiatric comorbidities, for children with various comorbid neurodevelopmental disorders and for individuals with high cognitive abilities or camouflaging strategies (Maddox et al., 2017; Morrier et al., 2017; Tillmann et al., 2018; Zander et al., 2016).

A particular research gap regarding the properties of the ADOS-2 and ASD assessment more generally exists for individuals with intellectual disability (ID) and/or sensory impairments (Molinaro et al., 2020; Sappok et al., 2013; Thurm et al., 2019).

ID is present in about 30% of children with ASD (Baio et al., 2018; Polyak et al., 2015) and among these children additional challenges such as sensory impairments and minimal verbal abilities might be present that complicate assessment (Molinaro et al., 2020; Szymanski et al., 2012). Still, diagnostic and intervention research of autism in minimally verbal individuals and those with sensory impairment is limited (Russell et al., 2019; Stedman et al., 2019). The latter is unfortunate, as inadequate or delayed identification of ASD in individuals with ID can lead to inappropriate provision of care and late access to community services with a negative impact on independence development (Baron-Cohen et al., 2009).

Impacting on the differential diagnosis and comorbidity assessment of ASD and ID and the accuracy of standardized diagnostic tools such as the ADOS-2, impairments in social functioning are part not only of the diagnostic operationalization of ASD but also ID, with social challenges increasing correlating with the severity of ID. According to DSM-5, the extent of social challenges must exceed those expected owing to level of ID to qualify as symptoms of ASD. However, the DSM-5 provides no further instructions about how or when ID may or may not explain symptoms of ASD.

The ADOS has several limitations when used with adults with ID; for instance, materials and activities may appear inappropriate for adults. For adults with ID, choosing an ADOS module based on verbal ability alone may not be suitable (Gotham et al., 2007). Therefore, some adaptations of the ADOS for use with individuals with ID have been suggested (Bal et al., 2020; Berument et al., 2005; Sappok et al., 2013). Bal et al. describe an adapted ADOS Module 1 (A-ADOS-M1) and Module 2 (A-ADOS-M2), designed for use with minimally verbal adolescents and adults retaining the original spirit of ADOS-2, but including new developmentally appropriate materials and tasks (Bal et al., 2020). The A-ADOS shows comparable sensitivity but improved specificity compared to ADOS-2 Modules 1 and 2.

Other less well established scales have been developed to assess ASD in minimally verbal adolescents and adults, such as the Music-Based Scale for Autism Diagnostics (MUSAD) (Bergmann et al., 2019), and interview-based screening measures like the Scale of Pervasive Developmental Disorder in Mentally Retarded Persons (PDD-MRS) (Kraijer & de Bildt, 2005). However, individuals with ID are likely not only to have limited verbal abilities but multiple disabilities such as additional sensory impairments affecting vision and hearing and other neurological conditions like epilepsy and complex movement disorders including dysarthria and apraxia of speech, all possibly affecting social communication. A particularly challenging group are individuals with hearing loss and associated communicative and social deprivation (Shefer et al., 2014). In order to consider a diagnosis of ASD in addition to ID and other disabilities to be meaningful in these cases, social communication and interaction challenges in this context need to be unexpected or significantly more impairing than expected in consequence of the individual’s developmental profile and general functional abilities. To enable a guided decision on the coexistence of ASD, clinicians need to collect and integrate all available information on the individual’s developmental history.

Evidence suggests that individuals with sensory impairments are at increased ASD risk (Hoevenaars-van den Boom et al., 2009; Rydzewska et al., 2019). For blind individuals, restricted symbolic play, increased stereotyped behaviours, difficulties in social interaction with peers and imitation and echolalic speech have been recurrently reported (Carvill, 2001; Rogers & Puchalski, 1986; Minter et al., 1991). These behaviors, referred to as “blindisms”, are to some extent explainable in the context of visual impairment (Andrews & Wyver, 2005) and are at the same time reminiscent of of ASD.

Comparable to visually impaired individuals, higher rates of autism have been reported in children who are deaf or hard of hearing (DHH) compared to their typically developing peers (Jure et al., 1991; Rosenhall et al., 1999; Szymanski et al., 2012). Mood and Shield (2014) investigated the clinical use of the ADOS-2 on eight DHH children who were primarily exposed to American Sign Language from birth and had been diagnosed with ASD previously. They concluded that although many of the core symptoms of ASD can be identified via clinical administration of the ADOS-2, the use of the instrument’s diagnostic algorithms to be inappropriate for this population. Individuals with early hearing loss are generally known to be at risk for additional disabilities. Approximately 40% of DHH children show additional disabilities, such as ID, ASD, visual disability and motor disorders (Cupples et al., 2018; Gallaudet Research Institute, 2011). This high rate is attributable especially to syndromic hearing loss or to medical conditions leading to multiple neurological impairments that include hearing loss (Kalatzis & Petit, 1998; Jure et al., 1991). The higher rates of ASD in individuals who are DHH are associated with higher rates of ID in this population in general (Gallaudet Research Institute, 2011: 9%; van Naarden et al., 2015: 23%; Holzinger et al., 2016: 13%). Moreover, it is the level of cognitive impairment and not the degree of hearing loss that is associated with ASD severity (Jure et al., 1991). For non-syndromic hearing loss (about 60% of congenital hearing loss) the genetic basis is identified for up to 70% of the cases (Satterfield-Nash et al., 2020) and does not show any overlap with the far more complex polygenetic architecture of ASD, making it difficult to suggest that deafness increases the risk for autism in general (Szymanski et al., 2012). Sensory deprivation per se as causal for ASD is considered refuted (Zafeiriou et al., 2007). It is also well known that it is not hearing loss itself but the possibly associated communicative deprivation that is associated with disruptive behavior, attention difficulties, problems with social cognition and socialization as well as stereotypical behaviors (Donno et al., 2010; Fellinger et al., 2012; Im-Bolter et al., 2006; Stevenson et al., 2010). Behaviors due to communication deprivation possibly in combination with aversive childhood experiences (bullying, mobbing, disturbed parent–child interactions) may at first sight resemble ASD symptoms and thus lead to and an overestimation of autism. On the other hand, autistic symptoms may be interpreted as typical characteristics associated with deafness, ID and deprivation leading to underdiagnosis of autism.

Despite the interest in measuring ASD symptoms in this group, existing standardized measures of ASD symptoms, foremost the ADOS-2 and Autism Diagnostic Interview-Revised in their current form, are considered as inappropriate for individuals with sensory impairments (Lord et al., 2012; Risi et al., 2006; Rutter et al., 2003).

Therefore, the objective of this study was to (a) to describe adaptations of the ADOS-2 for the assessment of DHH adults with ID and (b) to investigate the feasibility and reliability of this adapted measure by administering it in a complete sample of deaf adults with ID living in three therapeutic communities.

## Methods

### Adaptation of the ADOS-2

We sought to adapt the ADOS-2 (M1, M2, M3) for use with DHH adults with ID with signed language as their primary or only mode of communication including those with minimal language skills. The *overarching goal* of the adaptation process was to retain the original character of the ADOS-2 that provides structured tasks to observe social communication and interaction, play and repetitive behaviors (Lord et al., 2012) using different levels of stimulations or prompts. Nevertheless, the adapted version should be appealing to adults so that cooperation and social interaction are encouraged in a more age-appropriate fashion. The necessary adaptations concern module selection, modifications of setting, tasks and materials to make the procedures appropriate for adults with visual-manual communication as well as scoring modifications.

According to the manual’s guidelines, *module selection* is based on age and the level of expressive spoken language as outlined above. There is good evidence (e.g. Newport & Meier, 1985) that structural and functional milestones of sign language acquisition are very similar to those of spoken languages (e.g. age at appearance of first signs/words, combinations of signs/words, independent and complex clauses). Therefore, module selection was based on the complexity of the primary communication system, that for most profoundly deaf adults with ID is signed rather than spoken language. We administered M1 in individuals who did not use productive combinations of three signs including verbs. M2 was used in our study with participants who demonstrated combinations of at least three signs. M3 was administered if individuals demonstrated fluency in signed language by use of a variety and combinations of clauses to express communicative functions exceeding the “here and now” of the communication situation without necessarily demonstrating complete morpho-syntactic command.

### Assessment Setting

Caregivers or parents were not required to be present during the administration of the ADOS-2 in the adapted version. When caregivers accompanied the testee, they were seated next to him/her to allow for visual communication in order to contribute to his/her sense of security. However, as in the standard ADOOS-2, caregivers were asked not to interfere with the interactions between the examiner and the subjects. The presence of caregivers familiar with the subject’s expressive communication can be helpful for interpreting idiosyncratic signs or gestures for the examiners. All the activities were conducted sitting at a table so that the subjects feel as safe and comfortable as possible.

### Materials

In the adaptation, materials from ADOS-2 M3 were considered to be more appropriate for adults and therefore used in the “free play” task of M1 and M2 as well as in the “response to joint attention” task of M1 (Sappok et al., 2013), in addition to the materials from M1 and M2. Subjects were invited to choose materials according to their own preference. Likewise, picture books and pictures from M3 were offered in addition to the original materials of M1 and M2. Instead of the bubble gun duck, a regular wand was used to produce blowing bubbles as in our experience a bubble gun frequently causes a strong object relatedness with an impulse to manipulate the gun itself.

Additional adaptations of materials as suggested by Berument et al. (2005) had been used in in clinical practice preceding the study and not been found to be more acceptable to adults with deafness and ID as compared to the materials from M3. Therefore, they were only offered to the participants during the break. The A-ADOS module set for the assessment of minimally verbal adolescents and adults suggested by Bal et al. (2020) was not published at the time point this study started.

### Adaptations to Visual-Manual Communication

The administration of our adapted ADOS-2—particularly for M3—required high expressive and receptive sign language skills by the examiners and the ability to adapt visual communication to the communication level of the interlocutor (e.g. by adjusting the speed of communication, producing signs within the interlocutor’s line of sight, heightened use of body language, use of iconic signs and gestures). In addition, adaptation to the subjects’ primary signed communication system was necessary. Whenever any form of simultaneous communication (signing and speaking) was preferred by an individual, the examiner was required to adjust his/her communication mode. Since simultaneous exploration of objects or pictures and reception of signed language is not possible, the examiner had to be careful to allow for sufficient time for subjects to switch their attention between them and other topics of interest. During role plays with figures, the examiner had to ask for comments before or after a play action and offer support in holding the figures so that the individual could comment on what he/she was doing by use of manual communication.

### Modification of Activities

Response to Name (M1): The target group was composed of adults most of whom had a severe to profound sensorineural hearing loss. As a consequence, a response to their name presented verbally was not possible. On the other hand, it is not appropriate in Deaf culture to attain somebody’s attention by use of a name sign. Replacing the item by culturally accepted norms of attention getting (such as waving within an individual’s line of sight, tapping on the shoulder) was not considered to be comparable to the more specific response to one’s own name. Therefore, and as the item is not included in the diagnostic algorithm, “response to name” was deleted from the list of activities without replacement.

Joint Attention (M1, M2): During initial presses the sign LOOK followed by a head turn to the object of interest was presented in a neutral position without use of a directional element integrated into the sign. As a second step, the sign LOOK directed to the object—and thus fulfilling a similar function to a pointing gesture—was used and followed by a point.

Demonstration Task (M2): Evidently, a demonstration of an everyday routine by use of gestures (and facial expression) and the simultaneous explanation in a manual language is not possible. As the main objective of this task was the demonstration of routines by gestures and the use of gestures to represent objects the task was still performed. To help the subject understand the task and to avoid a narrative in sign language—with many signs being iconic in nature—the signs SHOW-me/THEATER/PANTOMIME was used in addition to drawing the contours of objects on the table and referring to the table as the bathroom. As a further prompt the imaginary toothbrush or towel was handed over to the subject followed by the request YOU and followed by SHOW-me/THEATER/PANTOMIME if necessary.

Functional Symbolic Imitation (M1, M2): The objects were named by signs (FROG, CAR, FLOWER, AIRPLANE, CUP). In national sign languages, some of the signs may represent the actions performed by or with them (e.g. AIRPLANE a Y-Handshape in a forward flying motion is used or the sign for CUP is produced by a hand movement toward the mouth with the index finger on the thumb as if holding a cup). Since labelling of the action to be performed with the objects should be avoided, the examiner took care to simply label the objects and not to label the actions (e.g. FLYING, DRINKING from a cup) by modifying the movement and/or simultaneous facial expression.

### Scoring Modifications

Overall level on Non-Echoed Spoken Language (M1, M2, M3): Complexity of expressive signed language rather than spoken language during the administration of the ADOS was scored. Spontaneous use of words was replaced by use of signs. Phrase speech referred to the use of three of more signs per utterance. Mostly correct use of sentences including complex sentences included a good command of sign language grammar (syntax, use of grammatical space, use of classifiers, combinations of clauses).

Frequency of Spontaneous Vocalizations Directed Towards Others (M2)*:* Scored as frequency of spontaneous signing/gesturing and vocalizations directed towards others.

Intonation of Vocalizations or Verbalizations (M1, Speech Abnormalities associated with Autism (M2,M3): Interpreted with regard to visual intonation in sign language (Dachkovsky & Sandler, 2009), abnormal size or location of signs (e.g. in periphery of usual signing space) and fluency of signing.

Echolalia (M1, M2, M3): Scored as exact repetition of the examiner’s signs or sign phrases.

Idiosyncratic/Stereotyped language (M1, M2, M3): In addition to highly repetitive signed phrases with consistent intonation patterns, signed neologisms and unusual palm orientations as well as use of pronouns were scored.

Gestures/ Descriptive, Conventional, Instrumental or Informative gestures (M1, M2, M3): Gestures describing the shape or manipulation of an object were scored here even though they might include Sign language classifiers (handshapes referring to classes of objects). The exclusive use of conventional signs containing classifiers was not counted as descriptive gesture.

Facial Expression Directed Towards Others (M1, M2, M3): As sign language uses facial expressions for lexical as well as grammatical functions (e.g. negations, questions) only facial expressions that communicate affect were scored.

Response to name (M1, M2): This item was deleted without replacement (see above).

### Additional Measures

#### Cognitive Assessment

Nonverbal intelligence was measured by the SON-R 2 ½-7 (Tellegen & Laros, 2007) or the SON-R 6-40 (Tellegen et al., 2012). Since the SON-R 2 ½-7 had to be used for clients who were not able to accomplish the simplest tasks of the SON-R 6-40. Therefore, age of reference was used as measure for nonverbal cognition in the following.

#### Feasibility

Three dimensions of *feasibility* (Bowen et al., 2009) were distinguished. Acceptability, i.e. the reaction of the involved individuals to the assessment was measured by the rate of those undergoing the full procedure. In addition, the ADOS-2 rapport scorings were compared to those of the original ADOS-2. Practicality was operationalized by the extent to which it was possible to administer the adapted ADOS within the regulatory time constraints of clinical examinations. Adaptation refers to the degree to which materials, activities, communication during the assessment and scoring had to be modified to appropriately administer the ADOS.

### Participants and Study Setting

All participants were recruited from three therapeutic living communities specialized for DHH adults with intellectual and multiple disabilities. The communities ensure access to communication by providing visual communication (mainly signed language) in the working and living environments. About one quarter of the staff is deaf. The sensitive milieu therapy approach focuses on the enhancement of social communication skills throughout the day. Staff is continually trained in responsive interaction and strategies to facilitate the residents’ active participation in a rich variety of everyday social interactions.

After exclusion of those with (i) a nonverbal IQ above 80, (ii) a less than moderate degree of hearing loss and (iii) a dual sensory impairment (deafblindness), 56 individuals and their respective legal guardians (if applicable) were invited to participate in the study. All of them gave their consent. The study was approved by the Ethics commission “Barmherzige Schwestern und Barmherzige Brüder” (EKB 14/18; 14.01.2019).

The sample had a mean age of 44.6 years (range 17 to 75, SD = 18.7) and a majority was male (65%). The level of intellectual functioning varied. However, a majority had a cognitive level of functioning above an age of reference (AOR) of 6 years (74%). A group of 9 participants with a nonverbal IQ below average (70–80) was included. The remaining subsample of those with cognitive AOR below 6 years was small (n = 6). The degree of hearing loss was severe or profound for all of the participants except one. Additional sample characteristics are provided in Table 1.

**Table 1:** Participant characteristics

	Module 1 (n = 8)	Module 2 (n = 16)	Module 3 (n = 32)
Age M (SD)	41.25 (19.91)	37.00 (18.52)	49.00 (17.48)
Sex male n (%)	4 (50.0)	14 (87.5)	19 (59.4)
Cognitve functioning AOR M (SD)	3.28 (1.58)	4.80 (1.39)	8.48 (9.72)
AOR > 9 < years (IQ71-80)	0 (0.0)	3 (18.8)	6 (18.8)
AOR 6-9years	5 (62.5)	10 (62.5)	26 (81.3)
AOT 3-6years	2 (25.0)	3 (18.8)	0 (0.0)
AOR > 3years	1 (12.5)	0 (0.0)	0 (0.0)
Hearing loss n (%)			
Moderate	0 (0.0)	0 (0.0)	1 (3.1)
Severe	0 (0.0)	1 (6.3)	5 (15.6)
Profound	8 (100.0)	15 (93.8)	26 (81.3)
Cerebral palsy n (%)	4 (50.0)	2 (14.3)	7 (21.9)
Epilepsy n (%)^a^	5 (62.5)	6 (42.9)	4 (12.5)
Psychiatric diagnosis n (%)	1 (12.5)	3 (21.4)	10 (32.3)

^a^significant differences (p < 0.05) between modules (Non-parametric One-way Anova for continuous variables and exact Fisher tests for categorical variables)

M1 was administered with 8 (few to no words (FNW): n = 5; some words (SW): n = 3), M2 with 16 and M3 with 32 participants. As expected in a sample of adults with a variety of mental ages mean, nonverbal cognitive AOR was significantly associated with the selected module (Non-parametric One-way ANOVA: χ^2^(2) = 25.2, p < 0.001), with the lowest scores in M1 and the highest in M3. Moreover, epilepsy was more prevalent in M1, especially compared to M3.

The adapted ADOS for DHH individuals with ID was applied as part of a research project evaluating two autism screening instruments in this special population (Hofer et al., 2021). All the 56 residents of the ‘Lebenswelt’ living community who met the inclusion criteria were assessed regardless of the screening results. The clinical examiners performing the adapted ADOS-2 were blinded for the screening results. Both of them, a clinical linguist (first author) and a neurologist and neuropediatrician (last author), are highly experienced in the field of hearing loss and ASD. They have been trained in the administration of the ADOS-2 and are fluent in Austrian Sign Language. The majority of assessments were performed in the developmental medicine outpatient clinic of the Hospital of St. John of God Linz. About one third of the participants were visited and assessed in their place of residence in a distraction-free room of the group home. Most of the participants were already known to at least one of the examiners. A caregiver from their therapeutic community was present in the room when examinations were conducted at the hospital. The two examiners performed all the assessments together with one of them directly interacting with the subject and the other in an observing role. Both examiners took notes during administration and independently scored the adapted ADOS-2 behavioral codes immediately afterwards. Finally, the two examiners arrived at a consensus opinion regarding clinical DSM-5 diagnoses (American Psychiatric Association, 2013) based on the ADOS-2 results and on comprehensive available data including information on cognitive and adaptive skills, informal observations of language and communication skills and on each subjects’ life history. Thus, diagnoses were not made independently of the adapted ADOS-2 results.

### Statistical Analysis

We conducted reliability analyses, focusing on both internal consistency and interrater reliability. To judge internal consistency, we estimated Cronbach’s Alpha and item-total (domain) correlations (i.e. the correlation of a single item with the domain score after exclusion of the item) separately for both raters. We conducted interrater-reliability analyses on item level, domain level (for the SA and RRB scores) and for the diagnosis derived from the total scores. On item level we estimated weighted kappa. On domain level, we computed the intraclass correlation (ICC) for absolute agreement between two raters based on a two-way mixed model (Gisev et al, 2013; Koo & Li, 2016). To judge interrater reliability for the diagnosis, we used kappa. Interrater reliabilities were graded as follows: (1) kappa (see Gisev et al., 2013), 0.00–0.20 = slight, 0.21–0.40 = fair, 0.41–60 = moderate, 0.61–0.80 = substantial and 0.81–1.00 = almost perfect. (2) ICC (see Koo & Li, 2016), < 0.50 = poor, 0.50–0.75 = moderate, 0.76–0.90 = good, > 0.90 = excellent. With regard to (diagnostic) validity, we computed correlations of items and domain scores with the final consensus diagnosis, and with other variables (verbal AOR, age) and estimated diagnostic accuracy statistics (sensitivity, specificity, positive predictive value and negative predictive value). Interrater reliability analyses were conducted using the irr package in R (Gamer et al., 2012). All other analyses were conducted using SPSS 26.

## Results

### Feasibility

#### Acceptability

The rate of acceptance of the adapted ADOS-2 by the participants was used as an indicator of acceptability. Due to the playful, conversational, and non-demanding character of the assessment and the adaptation to the communication needs of the participants, application in full was possible with almost all subjects (98%). The assessment of one participant with profound intellectual disability had to be terminated ahead of schedule due to behavior problems, but scoring was still possible due to sufficient observation time and observation situations.

As reported, materials of M3 were used in addition to the original materials of M1 and M2. As the examiners followed the individual interests of the subjects the adapted choice of materials offered did not lead to withdrawal or refusal even though individual subjects clearly expressed their disinterest in some of the materials. About half of the subjects reacted confused to or felt uncomfortable with the initial sequences of the Make-Believe Play Task, as most of the adults did not have experiences (currently or in the past) playing with figures. However, when the examiners took the initiative and modelled play actions or initiated interaction with the subject’s play figure various degrees of reciprocity and enjoyment as well as extension of the interaction could be observed as intended by this activity.

ADOS-2 results on overall quality of rapport can be interpreted as indication of acceptance of the assessment procedure by participants. Both raters on all three modules of the adapted version scored participants who were not diagnosed with ASD with 0 or 1 on rapport, not indicative of an unusual interaction or consistently uncomfortable situation. Of those finally diagnosed with ASD (n = 9) severely restricted rapport (scoring of 2 or higher) was reported in three of them.

Practicality can be understood as the extent to which the adapted procedure could be delivered within the context of usual clinical routines of autism assessment. In 98% (55 of 56), the assessment could be finalized within one appointment. In 1 patient the assessment had to be terminated due to behaviour problems. The execution time of the adapted ADOS-2 was available from video recordings that -for practical reasons—could only be completed for 32 of the participants. Duration of assessments varied from a minimum of 11 min (ADOS-2 M1) to a maximum of 60 min with an average of 28 min (SD = 11.5). Time required for administration was highest for M3 (M = 31.6 min, SD = 12.3), followed by M2 (M = 25.3 min, SD = 8.3) and M1 (M = 18.9 min, SD = 8.9). Administration time did not differ significantly (Mann–Whitney U = 61, p > 0.05) between individuals with an autism diagnosis (M = 30.2, SD = 17.8) and without a diagnosis (M = 27.4 min, SD = 10.5). The duration of the assessment can therefore be considered as appropriate for the target group.

#### Adaptation

As described in the methodology section all activities except “Response to Name” could be performed with the subjects with deafness and ID. Adaptations to deafness and intellectual disability concerned mainly the selection of the appropriate modules, the setting, the materials and visual-manual communication. In addition to the adaptation of communication to signed modality more general principles of visual communication such as allowing for sufficient time to switch attention between looking at picture materials or manipulating figures and the examiner had to be respected. Play with figures even required assisting the subjects with holding the figures to facilitate manual comments or direct speech that can easily be expressed simultaneously in spoken language. Only a small number of activities required modifications in implementation and scoring.

### Reliability Assessment

#### Internal Consistency

For M1-FNW (n = 5), three item-domain correlations for SA were negative. Consequently, Cronbach’s alpha was low (α_rater1_ = 0.26 and α_rater2_ = 0.38, respectively). For RRB, results were somewhat better. Cronbach’s alpha was 0.72 (rater 1) and 0.71 (rater 2), respectively. But again, there was a negative item-domain correlation. In contrast, internal consistency for M1-SW (n = 3) was virtually perfect. For SA and RRB, item-domain correlations *r*_*it*_ ranged from 0.55 to 1.00 and Cronbach’s alpha was 0.97 (rater 1) and 1.00 (rater 2) for SA and 0.96 (rater 1) and 0.96 (rater 2) for RRB, respectively.

For M2, item-domain correlations *r*_it_ (Table 2) ranged from 0.46 to 0.92 for SA with an excellent internal consistency (α_rater1_ = 0.90 and α_rater2_ = 0.93, respectively). For RRB, the item “Unusual sensory interests” did not correlate with the domain score (*r*_*it*_ = − 0.04 and − 0.18 for rater 1 and rater 2, respectively). Item-domain correlations for the remaining RRB items ranged from *r*_*it*_ = 0.58 to 0.92. Despite the negative item-domain correlation for one item, Cronbach’s alpha for the RRB domain was still acceptable (α_rater1_ = 0.76 and α_rater2_ = 0.77).

**Table 2:** Item domain correlations and Interrater reliability by modules

	Rater 1			Rater 2			Interrater		Correlation with Diagnosis
	M	SD	r_it_	M	SD	r_it_	Kappa^a^	Agreement	r_pb_
Module 2									
SA									
Pointing	0.19	0.54	0.849	0.19	0.54	0.846	1.000	100.0	0.942
Descriptive, conventional, instrumental, or informational gestures	0.13	0.34	0.576	0.19	0.40	0.465	0.765	93.7	0.381
Unusual eye contact	0.38	0.81	0.692	0.38	0.81	0.609	1.000	100.0	0.787
Facial expressions directed to others	0.38	0.62	0.81	0.50	0.63	0.737	0.789	87.5	0.689
Shared enjoyment in interaction	0.5	0.63	0.797	0.44	0.63	0.844	0.896	93.7	0.651
Showing	0.44	0.81	0.775	0.50	0.82	0.763	0.736	81.2	0.762
Spontaneous initiation of joint attention	0.13	0.34	0.846	0.25	0.45	0.455	0.600	87.5	0.882
Quality of social overtures	0.38	0.50	0.604	0.56	0.63	0.426	0.444	75.1	0.447
Amount of reciprocal social communication	0.75	0.58	0.717	0.94	0.44	0.654	0.607	81.2	0.541
Overall quality of rapport	0.56	0.73	.924	0.50	0.73	0.876	0.733	81.2	0.826
Cronbachs alpha	0.932			0.903					
RRB									
Stereotyped/idiosyncratic use of words or phrases	0.38	0.62	0.688	0.31	0.6	0.581	0.881	93.7	0.755
Unusual sensory interest in play material/person	0.13	0.34	− 0.182	0.19	0.4	− 0.044	0.765	93.7	− 0.173
Hand and finger and other complex mannerisms	0.19	0.54	0.902	0.25	0.58	0.923	0.837	93.7	0.915
Unusually repetitive interests or stereotyped behaviors	0.19	0.54	0.902	0.25	0.58	0.923	0.837	93.7	0.915
Cronbachs alpha	0.755			0.77					
Module 3									
SA									
Reporting of events	0.56	0.67	0.513	0.56	0.62	0.636	0.515	68.7	0.622
Conversation	0.81	0.59	0.721	0.63	0.55	0.647	0.568	81.3	0.574
Descriptive, conventional, instrumental, or informational gestures	0	0	0	0.03	0.18	0.394	0.000	96.9	0.475
Unusual eye contact	0.25	0.67	0.620	0.25	0.67	0.576	1.000	100.0	0.714
Facial expressions directed to examiner	0.25	0.44	0.729	0.25	0.44	0.733	1.000	100.0	0.655
Shared enjoyment in interaction	0.25	0.44	0.797	0.25	0.51	0.777	0.840	93.8	0.734
Quality of social overtures	0.38	0.66	0.593	0.5	0.57	0.505	0.587	74.9	0.553
Quality of social response	0.38	0.49	0.605	0.53	0.57	0.615	0.353	65.6	0.578
Amount of reciprocal social communication	0.59	0.71	0.826	0.59	0.67	0.794	0.820	90.6	0.680
Overall quality of rapport	0.25	0.44	0.797	0.25	0.51	0.716	0.840	93.8	0.734
Cronbachs alpha	0.888			0.89					
RRB									
Stereotyped/idiosyncratic use of words or phrases	0.06	0.25	0.510	0.19	0.47	0.507	0.458	90.6	0.772
Unusual sensory interest in play material/person	0.06	0.35	0.588	0.06	0.35	0.339	1.000	100.0	0.475
Hand and finger and other complex mannerisms	0	0	0	0	0	0	–	100.0	–
Excessive interest in or references to unusual or highly specific topics or objects or repetitive behaviors	0.16	0.45	0.309	0.25	0.51	0.472	0.732	90.6	0.567
Cronbachs alpha	0.554			0.552					

*r*_*it*_ item-domain correlation, *r*_*pb*_ point-biserial correlation

^a^Weighted Kappa

Finally, for M3 item-domain correlations (Table 2) ranged from 0.51 to 0.83 for SA with one exception. M (and SD) of the “Gestures” item was zero for rater 1, and thus *r*_*i*t_ could not be calculated. For rater 2, M and SD of the “Gestures” item were also small. However, the item-domain correlation (0.39) was still acceptable. Internal consistency was high for SA. Cronbach’s alpha for both raters was 0.89. For RRB, item-total (domain) correlations ranged from 0.31 to.59 with the exception of the “Mannerisms” item, with M = 0 (and SD = 0) for both raters. Internal consistency was poor for RRB (α_rater1/rater2_ = 0.55).

Overall, internal consistency was high to excellent for SA and lower for RRB. Internal consistency for SA was high to excellent for SA. For RRB, however, Cronbach’s alpha was lower, and for M3 even poor.

#### Interrater Reliability

Interrater agreement for the 12 out of 16 items used for M1-FNW and M1-SW was substantial to perfect (Kappas ranged from 0.68 to 1.00). For two items, Kappa was fair (0.41 and 0.60), while for the two remaining items, Kappas indicated only slight agreement (*Pointing*: Kappa = 0.20, *Gestures*: Kappa = 0.33).

For twelve out of 14 M2 items, Kappas exceeded 0.61, indicating substantial to almost perfect agreement. The remaining items show moderate agreement (Kappa > 0.44). Percent agreement varied from 75.1 to 100.0% with a median of 93.7%.

For seven out of 14 M3 items, Kappas exceeded 0.61, thus indicating a substantial to almost perfect agreement. Further four items showed moderate agreement (Kappas ranging from 0.46 to 0.59). *Quality of Social Response* had a Kappa of 0.35, indicating only fair agreement. Moreover, *Gestures* and *Mannerisms* had Kappas equal 0, however, this was due to M = 0 (and SD = 0). The exact agreement for M3 items ranged from 65.6 to 100% with a median of 92.2%.

Intraclass correlations (ICC) for SA, RRB, and total scores are reported in Table 3. For M2 and M3, ICCs were 0.94 and 0.96 for SA, 0.95 and 0.73 for RRB, and 0.96 and 0.95 for the total score. Interrater reliability (unweighted Kappa) for diagnoses directly derived from the ADOS-2 algorithms was substantial for M2 (Kappa = 0.77) and almost perfect for M3 (Kappa = 1.00). Percent agreement was 95% for M2 and 100% for M3. For M2, one participant classified as ASD by rater 2 just remained directly below the cut-off with rater 1.

**Table 3:** Interrater reliability by domain scores, total scores and modules

	Module 2 (n = 16)			Module 3 (n = 32)		
	Rater 1	Rater 2	Interrater reliability	Rater 1	Rater 2	Interrater reliability
SA M (SD)	3.938 (4.754)	4.625 (4.530)	0.935^a^	3.719 (3.787)	3.781 (3.858)	0.964^a^
RRB M (SD)	0.875 (1.586)	1.000 (1.673)	0.953^a^	0.281 (0.813)	0.581 (1.119)	0.726^a^
Total M (SD)	4.813 (6.167)	5.625 (5.886)	0.958^a^	4.031 (4.344)	4.250 (4.925)	0.946^a^
Diagnosis n (%)						
No	14 (87.5)	13 (81.3)	93.7/0.771^b^	26 (81.3)	26 (81.3)	100.0/1.000^b^
ASD	0	1 (6.3)		2 (6.3)	2 (6.3)	
Autism	2	2 (12.5)		4 (12.5)	4 (12.5)	

^a^Intraclass correlation

^b^First value = % agreement, second value = kappa

### Validity

#### Item Correlations with ASD Diagnosis

M2 item correlations (point-biserial correlations, *r*_*pb*_; see Table 2) with autism classification (ASD vs. no ASD) were all above *r*_*pb*_ = 0.45 for SA except for *Gestures* (*r*_*pb*_ = 0.38). For RRB, there was a negative correlation between ASD classification and *Unusual Sensory Interests* (*r*_*pb*_ = − 0.17). The remaining correlations for RRB were 0.76 or higher. For M3, item diagnosis correlations ranged from 0.48 (*Gestures*) to 0.77 (*Stereotyped Use of Words*).

#### Item and Domain Correlations with Patient Characteristics

Item and domain correlations with chronological age and nonverbal cognitive age are shown in Table 4. Although there were some moderate correlations (r > 0.30) for M2 none of them was significant. For M3, we found some significant correlations. Cognitive age correlated with *Shared Enjoyment in Interaction* (r = 0.38, p < 0.05), *Overall Quality of Rapport* (r = 0.37, p < 0.05) and *Extensive Interest* (r = 0.38, p < 0.05). Chronological age was negatively associated with *Quality of Social Response* (r = 0.41, p < 0.05).

**Table 4:** Item and domain correlations with chronological and cognitive age

Module 2			Module 3		
	Chron. age	Cogn. age		Chron. age	Cogn. age
*SA total score*	0.113	− 0.023	*SA total score*	− 0.299	0.151
Pointing	0.071	0.143	Reporting of events	− 0.281	0.033
Descriptive, conventional, instrumental, or informational gestures	− 0.14	− 0.184	Conversation	− 0.306	0.117
Unusual eye contact	− 0.07	0.076	Descriptive, conventional, instrumental, or informational gestures	− 0.106	− 0.008
Facial expressions directed to others	0.282	− 0.028	Unusual eye contact	− 0.163	− 0.026
Shared enjoyment in interaction	0.334	0.064	Facial expressions directed to examiner	− 0.165	− 0.055
Showing	0.198	0.019	Shared enjoyment in interaction	− 0.225	0.384*
Spontaneous initiation of joint attention	0.029	0.091	Quality of social overtures	− 0.299	0.073
Quality of social overtures	− 0.046	− 0.166	Quality of social response	− 0.409*	0.072
Amount of reciprocal social communication	0.007	− 0.372	Amount of reciprocal social communication	− 0.209	0.144
Overall quality of rapport	0.231	0.103	Overall quality of rapport	− 0.181	0.373*
*RRB total score*	− 0.015	0.070	*RRB total score*	− 0.290	0.094
Stereotyped/idiosyncratic use of words or phrases	− 0.08	− 0.061	Stereotyped/idiosyncratic use of words or phrases	− 0.144	− 0.007
Unusual sensory interest in play material/person	− 0.025	− 0.052	Unusual sensory interest in play material/person	− 0.096	0.011
Hand and finger and other complex mannerisms	0.029	0.153	Hand and finger and other complex mannerisms	–^a^	–^a^
Unusually repetitive interests or stereotyped behaviors	0.029	0.153	Excessive interest in or references to unusual or highly specific topics or objects or repetitive behaviors	− 0.241	0.383*

^a^Item had a zero variance

*p < 0.05

#### Sensitivity and Specificity

As shown in Table 5 sensitivity and specificity of the adapted ADOS-2 using the ADOS-2 scoring algorithms was high. However, due to the small number of ASD diagnoses and the clinical diagnoses not made independently of the adapted ADOS, results need to be interpreted with caution.

**Table 5:** Diagnostic validity—ADOS vs. consensus diagnosis

		Sensitivity (%)	Specifity (%)	PPV (%)	NPV (%)
Autism vs. ASD/no					
M2 (n = 16)	Rater 1	100.0	93.3	50.0	100.0
	Rater 2	100.0	93.3	50.0	100.0
M3 (n = 32)	Rater 1	100.0	100.0	100.0	100.0
	Rater 2	100.0	100.0	100.0	100.0
Total M1-M3 (n = 56)	Rater 1	100.0	96.0	75.0	100.0
	Rater 2	100.0	100.0	100.0	100.0
Autism/ASD vs. no					
M2 (n = 16)	Rater 1	100.0	100.0	100.0	100.0
	Rater 2	100.0	92.9	66.7	100.0
M3 (n = 32)	Rater 1	100.0	92.9	66.7	100.0
	Rater 2	100.0	92.9	66.7	100.0
Total M1-M3 (n = 56)	Rater 1	100.0	91.5	69.2	100.0
	Rater 2	100.0	93.6	75.0	100.0

*PPV* positive predictive value, *NPV* negative predictive value

## Discussion

Modules 1, 2 and 3 of the ADOS-2 were developed to assess ASD symptoms in DHH adults with ID. Necessary adaptations—all to a limited extent—concerned selection of the appropriate module, requirements of visual communication, test materials, activities and scoring. Practicability of the measure, particularly acceptance by the participants and time-economic delivery was high. It was possible to fully assess almost all of the residents (98%) of three therapeutic housing communities for DHH adults with ID and to derive scores for both ADOS-2 categories of social affect and restricted and repetitive behaviors for all of them. The high acceptability demonstrates the appropriateness of the used materials. However, as the A-ADOS by Bal et al. (2020) was published after the start of our study their proposed adaptations could not be integrated in our study design. Especially for ADOS-2 M1 the suggestions by Bal et al. could be a reasonable complement and their suggested materials could contribute to a longer implementation period.

Internal consistency was found to be excellent for the social affect domain of M2 and good for M3, whereas it was acceptable for restricted and repetitive behaviors of M2 and poor for M3. This finding does not seem to be specific to adult age, ID or deafness, as the internal consistency stated for the US standardization sample of the ADOS-2 is good for social affect and lower for the RRB scale (M1–M3: r = 0.51–0.66). For M1 the number of participants was too small to derive reliable results.

Correlations between individual items and the total domain score for M2 were at a minimum of r = 0.43 with the exception of one SA item (*Gestures*) and one RRB item (*Unusual Sensory Interests*). The finding of less atypicality in the use of gestures is most likely a characteristic of the DHH participants living in a community with consistent use of signed communication and a focus on the enhancement of manual communication. Similarly, we found less atypicality in the initiation of joint attention as compared to the ADOS-2 children’s norms. Again, *initiation of joint attention* -particularly by pointing—is essential in visual communication and a target of communication intervention in DHH individuals with ID. The low correlation of unusual sensory interests with other RRB items is unexpected. The very few incidents observed during the assessment might be a consequence of adult age and of interventions aiming for the reduction of socially inappropriate behaviors.

In M3, only two items (*Gestures* and *Quality of Social Response*) scored lower than 0.5 for the item-subdomain correlation. As with M2 we assume effects of the visual communication environment and of social interventions.

Good internal consistency for SA and lower internal consistency for RRB found in our sample of individual who are DHH have also been reported for samples of minimally verbal adolescents and adults (Bal et al., 2020) and for children (Lord et al., 2012).

Interrater reliability as measured by Kappa scores was substantial for most items and at least moderate for the remaining ones, except for the M3 item *Quality of Social Response*. The relatively low agreement between raters (65.6%) for *Social Response* maybe due to different perceptions of abnormality by the two raters based on different lengths of experience in the field. Since reduced quality of social response can also be considered a typical consequence of communicative and social deprivation associated with deafness rating can be challenging.

Within our clinical procedures it was not possible to evaluate performance of the adapted ADOS-2 independent from clinical decision making about the ASD diagnosis. Therefore, the high sensitivity and specificity scores can only be regarded as first indications of high performance of the adapted measure.

Even though the study was performed with all the residents of three therapeutic living communities fulfilling the inclusion criteria, the small size of the study, particularly for M1, is a limitation. However, the sample seems to be similar to other populations living in institutions for adults with ID in terms of age, sex and distribution of ID (e.g. Sappok et al., 2013).

In conclusion, the current ADOS-2 adapted for adults who are DHH and have ID may close a gap in the measures for assessing symptoms of ASD in adults with ID and hearing loss who communicate primarily in the visual modality. The present study provides support for the feasibility of the adapted ADOS. Our results suggest that ASD symptoms can be reliably identified by administering the adapted ADOS. The used materials are original materials from ADOS-2 M1-3 and the scoring algorithms do not differ from the original ADOS-2 scoring. Since all activities in the adapted ADOS require flexible linguistic and nonverbal communication adaptation to the individual’s communication needs, excellent command of signed language (particularly for M3) and expertise in deafness, ID and ASD (not restricted to individuals with hearing loss) is deemed necessary for valid administration and interpretation. Despite of promising psychometric properties the adapted ADOS needs further validation in larger samples, in particular for M1. Since psychometric properties in many ways are similar to the ADOS-2 administered to children, the current adapted ADOS-2 may even be appropriate for use with children who are deaf and communicate in signed modality.
