# Correction to: Assessment of Autism Spectrum Disorder in Deaf Adults with Intellectual Disability: Feasibility and Psychometric Properties of an Adapted Version of the Autism Diagnostic Observation Schedule (ADOS-2)

**DOI:** 10.1007/s10803-021-05245-9

**Published:** 2021-08-17

**Authors:** D. Holzinger, C. Weber, S. Bölte, J. Fellinger, J. Hofer

**Affiliations:** 1 Forschungsinstitut für Entwicklungsmedizin, Johannes Kepler Universität Linz, Linz, Austria; 2 Institut für Sinnes- und Sprachneurologie, Konventhospital Barmherzige Brüder, Seilerstätte 2, 4021 Linz, Austria; 3 Institut für Sprachwissenschaft, Karl-Franzens-Universität Graz, Graz, Austria; 4 Institut für Inklusive Pädagogik, Pädagogische Hochschule OÖ, Linz, Austria; 5 Department of Women’s and Children’s Health, Center of Neurodevelopmental Disorders (KIND), Centre for Psychiatry Research, Karolinska Institutet & Stockholm Health Care Services, Region Stockholm, Stockholm, Sweden; 6 Child and Adolescent Psychiatry, Stockholm Health Care Services, Region Stockholm, Stockholm, Sweden; 7 Curtin Autism Research Group, Curtin School of Allied Health, Curtin University, Perth, WA Australia; 8 Abteilung für Sozialpsychiatrie der Universitätsklinik für Psychiatrie und Psychotherapie, Medizinische Universität Wien, Vienna, Austria; 9 Abteilung für Pädiatrie I, Medizinische Universität Innsbruck, Innsbruck, Austria

## Correction to: Journal of Autism and Developmental Disorders 10.1007/s10803-021-05203-5

Correction regarding Table 1 and the corresponding text passage under “Participants and Study Setting”. Corrections are highlighted bold.

**Table 1:** Participant characteristics

	Modul 1 (n = 8)	Modul 2 (n = 16)	Modul 3 (n = 32)
Age M (SD)	41.25 (19.91)	37.00 (18.52)	49.00 (17.48)
Sex male n (%)	4 (50.0)	14 (87.5)	19 (59.4)
Cognitive functioning AOR^a^ M (SD)	3.28 (1.58)	4.80 (1.39)	6.77 (1.73)
**Cognitive functioning AOR**^**a**^** n (%)**			
** ≥ 9 years**	**0 (0.0)**	**1 (6.3)**	**4 (12.5)**
** < 9 to 6 years**	**1 (12.5)**	**1 (6.3)**	**21 (65.6)**
** < 6 to 3 years**	**4 (50.0)**	**14 (87.5)**	**7 (21.9)**
** < 3 years**	**3 (37.5)**	**0 (0.0)**	**0 (0.0)**
Hearing loss n (%)			
Moderate	0 (0.0)	0 (0.0)	1 (3.1)
Severe	0 (0.0)	1 (6.3)	5 (15.6)
Profound	8 (100.0)	15 (93.8)	26 (81.3)
Cerebral Palsy n (%)	4 (50.0)	2 (14.3)	7 (21.9)
Epilepsy^a^ n (%)	5 (62.5)	6 (42.9)	4 (12.5)
Psychiatric Diagnosis n (%)	1 (12.5)	3 (21.4)	10 (32.3)

^a^significant differences (p < 0.05) between modules (Non-parametric One-way Anova for continuous variables and exact Fisher tests for categorical variables)

Participants and Study Setting (3rd paragraph)

The sample had a mean age of 44.6 years (range 17 to 75, SD = 18.7) and a majority was male (65%). The level of intellectual functioning varied. **While 50% had a cognitive level of functioning above an age of reference (AOR) of 6 years, in 50%the AOR was below 6 years.** The degree of hearing loss was….

